# Development and Testing of a Novel Measure to Assess Fidelity of Implementation: Example of the Mini-AFTERc Intervention

**DOI:** 10.3389/fpsyg.2020.601813

**Published:** 2020-11-25

**Authors:** Nathalie Georgia Brandt, Calum Thomas McHale, Gerald Michael Humphris

**Affiliations:** ^1^Division of Population and Behavioural Sciences, School of Medicine, University of St Andrews, St Andrews, United Kingdom; ^2^Edinburgh Cancer Centre, Western General Hospital, National Health Service Lothian, Edinburgh, United Kingdom

**Keywords:** fidelity, implementation, Mini-AFTERc intervention, breast cancer, fear of cancer recurrence

## Abstract

**Background:**

Fidelity of implementation (FOI) reflects whether an intervention was implemented in clinical practice according to the originally developed manual and is a key aspect in understanding intervention effectiveness. To illustrate this process of developing a fidelity measure, this study uses the Mini-AFTERc, a brief psychological intervention aimed at managing breast cancer patients’ fear of cancer recurrence, as an example.

**Objectives:**

To illustrate the development of an FOI measure through (1) applying this process to the Mini-AFTERc intervention, by including the design of a scoring system and rating criteria; (2) content validating the FOI measure using thematic framework analysis as a qualitative approach; (3) testing consistency of the FOI measure using interrater reliability.

**Methods:**

The FOI measure was developed, its scoring system modified and the rating criteria defined. Thematic framework analysis was conducted to content validate the FOI measure using nine intervention discussions between four specialist cancer nurses and four breast cancer patients, and one simulated breast cancer patient. Intraclass-correlation was conducted to assess interrater reliability.

**Results:**

The qualitative findings suggested that the Mini-AFTERc FOI measure has content validity as it was able to measure all five components of the Mini-AFTERc intervention. The interrater reliability suggested a moderate to excellent degree of reliability among three raters, r*_*ICC*_* = 0.84, 95% CI [0.51, 0.96].

**Conclusion:**

The study has illustrated the steps that an FOI measure can be developed through a systematic approach applied to the Mini-AFTERc intervention. The FOI measure was found to have content validity and was consistently applied, independently, by three researchers familiar with the Mini-AFTERc intervention. Future studies should determine whether similar levels of interrater reliability can be obtained by distributing written and/or video instructions to researchers who are unfamiliar with the FOI measure, using a larger sample. Employing developed and validated FOI measures such as the one presented for the Mini-AFTERc would facilitate implementation of interventions in the FCR field in clinical practice as intended.

**Clinical Trial Registration:**

www.ClinicalTrials.gov, identifier: NCT03763825.

## Introduction

**FIGURE 1:**
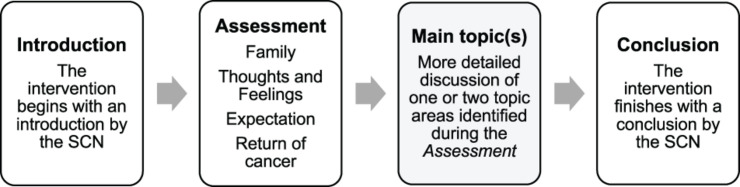
The procedure of the Mini-AFTERc intervention.

Fear of cancer recurrence (FCR) is the “Fear, worry, or concern about cancer returning or progressing” (Lebel et al., 2016, p. 3267). FCR is a common issue that up to 97% of cancer survivors experience and, importantly, 22–87% of cancer survivors reported experiencing moderate to high FCR levels (Simard et al., 2013). The level of FCR has been associated with the time since primary surgery, the type of surgery and treatment, having symptoms of pain, fatigue, unmet needs, and age (Simard et al., 2013). This paper focused on the Mini-AFTERc intervention which aims to support breast cancer survivors in the management of FCR and was developed for patients experiencing moderate FCR levels (Davidson et al., 2018; McHale et al., 2020). This intervention is based on the cognitive-behavioral therapy approach of Leventhal’s Self-Regulatory Model (Leventhal et al., 1984). Its purpose is to normalize breast cancer patients’ fears and concerns by addressing the primary causes of these fears (Lee-Jones et al., 1997; Davidson et al., 2018; McHale et al., 2020). The intervention consists of 5 key components: Assessment, Family, Thoughts and Feelings, Expectation, and Return of cancer (AFTERc; Figure 1). The Mini-AFTERc is a structured 30 min counseling intervention, which is designed to be delivered during a single telephone conversation led by a specialist cancer nurse (SCN), who has undertaken an intervention training course and has been provided with the intervention manual.

Fidelity of implementation (FOI), also referred to as fidelity of delivery, is an important yet understudied aspect of psychological interventions. FOI has been defined as the extent to which an intervention was delivered as intended, such that the manual originally developed was adhered to, and the critical components of the intervention were present (Orwin, 2000; Century et al., 2010). FOI helps to establish internal and construct validity by providing evidence for the extent to which the implementation of the intervention followed the intervention manual (Stains and Vickrey, 2017). Additionally, FOI contributes to the establishment of external validity by increasing credibility and confidence of scientific findings for practitioners and policymakers, and aids a better understanding of what constitutes an effective intervention (Borrelli, 2011; Brownson et al., 2017). As such, conclusive statements about intervention effects cannot be made without an assessment of FOI (Borrelli, 2011).

Measuring FOI enables researchers to confirm what exactly has been implemented and worked, and hence what can be replicated by research (Nelson et al., 2012). It also aids in identifying aspects of the intervention that were implemented poorly, which may guide future improvements (Nelson et al., 2012). Meta-analyses summarizing over 600 intervention studies targeting mental and physical health, highlighted that higher levels of intervention fidelity were associated with better treatment outcomes (Durlak and DuPre, 2008; Saini, 2009). These findings demonstrated that studies using structured intervention manuals and assessing FOI produced larger effect sizes than studies that did not.

Previous studies that have developed structured psychological interventions for FCR do not robustly or consistently address FOI using evidence-based measures (Lebel et al., 2014; Dodds et al., 2015; Dieng et al., 2016; Butow et al., 2017; van de Wal et al., 2017; Tomei et al., 2018). For example, the SWORD study by van de Wal et al. (2017) used a checklist for a group cognitive behavioral therapy for depression (Hepner et al., 2011), which was not developed specifically for the SWORD intervention. This checklist was developed for the Building Recovery by Improving Goals, Habits, and Thoughts (BRIGHT) and BRIGHT-2 interventions (Hepner et al., 2011). It is unclear to what extent this checklist is relevant and representative of the blended cognitive behavior therapy of the SWORD study, and whether it had been tested previously to ensure content validity. Furthermore, some of these studies do not report how FOI assessments were used to draw conclusions about the intervention, such that they have not been linked to the intervention’s effectiveness. A further example is the ConquerFear study by Butow et al. (2017) which reported that clinicians completed session checklists to ensure fidelity. Additionally, a random 11% of audio recorded intervention sessions was reviewed independently by one of the study team (a clinical psychologist) and feedback was provided to clinicians where non-fidelity was identified. The authors do not report any intra-rater reliability and it is unclear whether the checklist included the core components of the intervention.

Considering the importance of assessing FOI for the robust development and implementation of psychological interventions, it is essential to consistently test and improve fidelity with which current interventions are implemented in clinical practice. As existing measures of fidelity are often generalized to allow the assessment of intervention implementation across a variety of settings and interventions (Breitenstein et al., 2010), they are not always applicable to the intervention being studied. At present, a specific measure to assess FOI of the Mini-AFTERc intervention does not exist and thus necessitates development and testing.

The AFTER intervention (Humphris and Ozakinci, 2008), on which the Mini-AFTERc was based, stressed the need to attend to the therapeutic alliance. Experience of applying this intervention in clinical practice demonstrated that users of the intervention aspired greater flexibility to follow issues that transpired in the patient interaction. Additionally, it increased the chances of the interventionist to provide acknowledgment to patient difficulties and empathize with emotional expressions. Therapeutic alliance can be defined as the collaborative and affective relationship between the therapist and patient (Bordin, 1994; Luborsky, 1994). The therapeutic alliance is regarded to be the most significant aspect in attaining positive therapeutic change (Paul and Charura, 2014). Accordingly, it is important that the interventionist understands the principles of therapeutic alliance to facilitate a strong therapeutic relationship with their client (Paul and Charura, 2014). Earlier meta-analyses including 573 studies concerning youth and adult psychotherapy demonstrated a moderate but reliable link between good therapeutic alliance and positive intervention outcome (Shirk and Karver, 2003; Karver et al., 2006; Horvath et al., 2011; Shirk et al., 2011; Flückiger et al., 2018). Furthermore, a review by Ackerman and Hilsenroth (2003) including 25 studies investigated the type of therapist characteristics and techniques that positively impact on therapeutic alliance. They reported that personal attributes such as being warm and interested, and therapist techniques such as exploration and reflection, impact positively on therapeutic alliance (Ackerman and Hilsenroth, 2003). Therefore, these aspects should be considered when rating the level of fidelity for the Mini-AFTERc intervention and possibly any intervention involved with modifying FCR levels.

Assessing FOI is essential to draw correct conclusions about the effectiveness of the Mini-AFTERc intervention (Borrelli, 2011). It allows researchers to verify that the therapeutic approach used by the SCNs during the intervention represents the defined intervention, and aids in establishing internal, construct, and external validity (Borrelli, 2011; Brownson et al., 2017; Stains and Vickrey, 2017). Particularly, as there is continued work to develop the Mini-AFTERc intervention, close attention is required to devise a bespoke measure for a major trial. Therefore, a comprehensive FOI measure representing the flexibility of the Mini-AFTERc intervention should be developed. Importantly, when defining the rating criteria, therapeutic alliance should be considered (Ackerman and Hilsenroth, 2003). Lastly, interrater reliability of the novel FOI measure, which reflects the variation among two or more raters who measure the same groups of participants, should be established to allow its use in research or clinical applications (Koo and Li, 2016).

This study aimed to develop a comprehensive FOI measure for the Mini-AFTERc intervention and act as an example to other investigators wishing to assess the effectiveness of their interventions in the FCR field. The development of the FOI measure included the design of an unambiguous scoring system and rating criteria to categorize the level of fidelity, providing researchers with a standardized way to quantify how closely the SCNs adhered to the Mini-AFTERc intervention manual. The study objectives were to:

1. Develop a Mini-AFTERc FOI measure, including the design of a scoring system and rating criteria;
2. Content validate the Mini-AFTERc FOI measure using thematic framework analysis as a qualitative approach;
3. Test the consistency of the Mini-AFTERc FOI measure using interrater reliability.

## Materials and Methods

### Study Design

The qualitative study described in this paper was part of the Mini-AFTERc pilot trial (McHale et al., 2020). Thematic framework analysis was employed using an essentialist/realist approach (Terry et al., 2017) to address the study objectives. Thematic framework analysis was chosen due to its flexibility, as it can be used within most theoretical frameworks (Terry et al., 2017).

### Study Objective 1: Development of the Mini-AFTERc FOI Measure, Including the Design of a Scoring System and Rating Criteria

**FIGURE 2:**
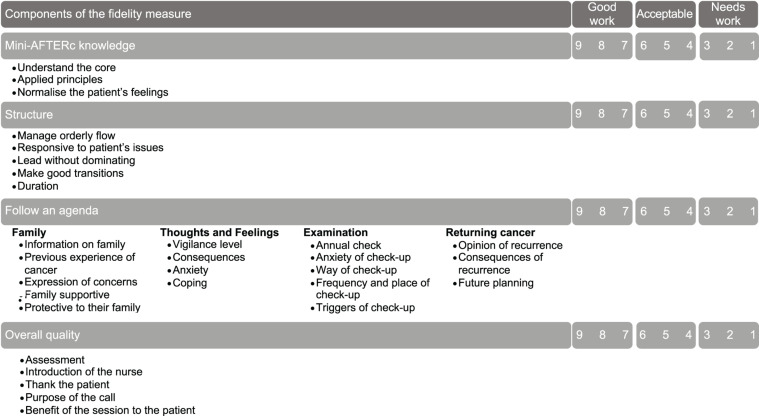
Initial version of the Mini-AFTERc FOI measure.

The FOI Rating System, designed by Forgatch et al. (2005), was used to inform the development of an initial version of the Mini-AFTERc FOI measure (Figure 2). This rating system was selected because it was developed to evaluate the fidelity of a manualized theory-based interventions through audio-visual recordings of the intervention being delivered, which fit well with the Mini-AFTERc pilot study protocol (Forgatch et al., 2005). Initial testing of the FOI measure with a small sample of audio recorded Mini-AFTERc intervention telephone calls, collected as part of a feasibility study (Davidson et al., 2018), highlighted that clarification and refinement of content, scoring, and rating was necessary.

#### Identification of Components

The current iteration of the FOI measure, presented in this paper, was developed by the first author (NGB). After studying the Mini-AFTERc intervention training manual and the initial FOI measure, NGB identified that, while the FOI measure addressed some fundamental aspects of the intervention, including Mini-AFTERc knowledge, structure, follow an agenda, and overall quality, it did not address the separate four core components: *Family*, *Thoughts and Feelings*, *Expectation*, and *Return of cancer*. Missing, or lacking details, of these essential components leads to an inability to address the intervention’s flexibility and comprehensiveness, thus limiting the ability to assess properly the intervention’s FOI. Hence, to allow the development of a comprehensive FOI measure specifically designed for the Mini-AFTERc intervention, the core components must be addressed separately to enable accurate scoring and rating.

#### Scoring of the Mini-AFTERc FOI Measure

The initial development of the FOI measure included a 9-point scoring system (Figure 2) which NGB redesigned to a 3-point scale. This 3-point scale of adherence was designed to reduce the ambiguity of the scoring process and allow raters to employ their discriminative abilities (Jacoby and Matell, 1971), reducing the possibility of introducing error. The possible adherences scores are as follows: (2) high adherence, (1) moderate adherence, and (0) low adherence. Adherence is defined as whether “a program service or intervention is being delivered as it was designed or written” (Mihalic, 2004, p. 2).

The intervention manual instructs that it is not feasible, within the allotted 30 min, to cover all four core intervention discussion topics in extensive detail. If it is apparent that a patient has potential issues in all four topics, the SCN should prioritize two topics and potentially offer another session. Therefore, the scoring system was designed with the expectation that only one to two core discussion topics would be the major focus of discussion during the intervention. At least half of the subcomponents must be addressed for a topic to be identified as a major focus of the intervention discussion. During the major topic discussion phase, at least half of the subcomponents had to be explicitly addressed by the SCN in at least one major discussion topic for “high adherence” to be scored, providing the SCN scored “high adherence” for all components. To reduce the possibility of scoring unfairly and/or inaccurately, high fidelity could be achieved regardless of whether half of the subcomponents of one main topic were addressed, or all subcomponents of two main topics (Table 1; parts 3.1–3.4).

**TABLE 1:** Possible adherence scores for each component and total fidelity score calculation.

Part no. and components	No. of sub-components	High adherence (2)	Moderate adherence (1)	Low adherence (0)
Part 1: Introduction	4	8	4	0
Part 2: Assessment of Family	–	2	1	0
Part 2: Assessment of Thoughts and Feelings	–	2	1	0
Part 2: Assessment of Expectation	–	2	1	0
Part 2: Assessment of Return of cancer	–	2	1	0
Part 3: Topic of which specific attention is required				
Part 3.1: Family*	6	6^a^–12^b^	3^a^–6^b^	0
Part 3.2: Thoughts and Feelings*	4	4^a^–8^b^	2^a^–4^b^	0
Part 3.3: Expectations*	6	6^a^–12^b^	3^a^–6^b^	0
Part 3.4: Return of cancer*	4	4^a^–8^b^	2^a^–4^b^	0
Part 4: Conclusion	3	6	3	0
Part 5: Duration	–	2	1	0
Total fidelity score range	–	28^c^–48^d^	14^c^–24^d^	0
Level of fidelity and score range		High 28–48	Moderate 14–27	Low 0–13

*Only rate the topic if discussed in greater detail, i.e., it was the major focus of the intervention. ^*a*^Score if half of the subcomponents were addressed. ^*b*^Score if all subcomponents were addressed. ^*c*^Score if one topic with four subcomponents was discussed in greater detail, of which two subcomponents were discussed. ^*d*^Score if two topics with six subcomponents were discussed in greater detail, of which all subcomponents were discussed.

#### Rating Criteria of the Mini-AFTERc FOI Measure

The decision to rate adherence as high (2) or moderate (1) was informed by principles positively contributing to therapeutic alliance (Ackerman and Hilsenroth, 2003; Table 2 and Supplementary File 1 for full principle definitions). If the SCN displayed personal attributes and used therapist techniques during the intervention (Table 2), the SCN should receive a rating indicating high adherence (2); otherwise, they should receive a rating indicating moderate adherence (1). Additionally, if the SCN did not consider the intervention’s flexibility, they should receive a rating indicating moderate adherence (1); this includes adhering too strictly to the manual and not considering possible issues that had been shared previously in the interaction by the patient. A rating of 0 should be attributed to a component that is not addressed.

**TABLE 2:** Principles positively contributing to therapeutic alliance.

Personal attributes	Therapist techniques
• Respectful	• Exploration
• Flexible	• Reflection
• Trustworthy	• Facilitates the expression of affect
• Warm	• Accurate interpretation
• Confident	• Attends to the patient’s experience
• Interested	• Supportive
• Honest	• Affirming
• Open	• Understanding
• Friendly	
• Alert	

**Adapted from “A review of therapist characteristics and techniques positively impacting the therapeutic alliance” by Ackerman and Hilsenroth (2003). See Supplementary File 1 for definitions.**

The researcher should rate the duration as 2 if it was within the limits of 25–35 min, 1 if it was between 20–25 and 35–40 min, and 0 if it was below 20 min or above 40 min. These limits were discussed and agreed on by the three researchers involved in this study, as duration is a key component in the design of the intervention. Lastly, no negative points should be given for flexibility; when rating the main topic of the intervention, points should be given for any subcomponents of the main topic already discussed during the *Assessment* process.

### Study Objective 2: Content Validate the Mini-AFTERc FOI Measure Using Thematic Framework Analysis

This FOI measure was tested by qualitatively analyzing the audio-recordings of the intervention discussions using thematic framework analysis (Terry et al., 2017).

#### Participants

**FIGURE 3:**
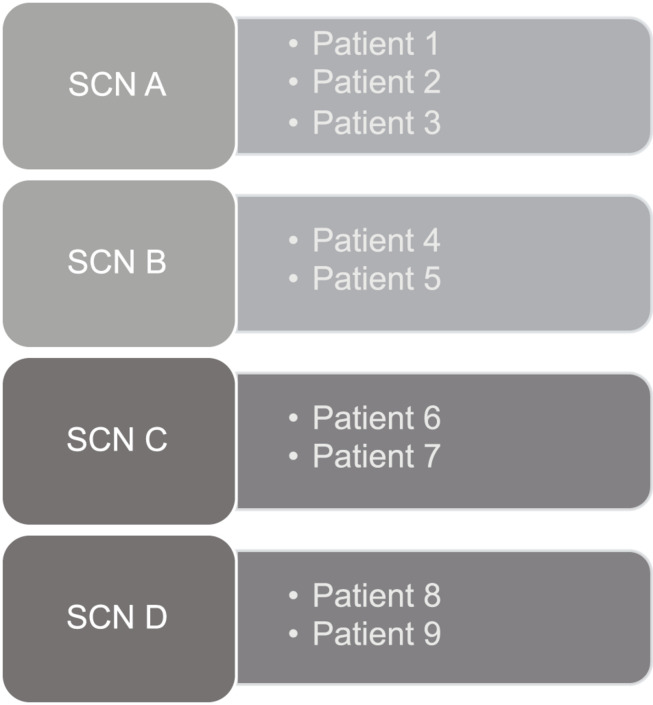
Mini-AFTERc intervention interviews between SCNs and breast cancer patients.

A convenience sample of nine audio-recorded Mini-AFTERc intervention discussions was used to test the FOI measure. Four SCNs (A-D) from two breast cancer centers in National Health Service (NHS) Scotland, five breast cancer patients and one simulated breast cancer patient produced these nine intervention discussions. SCN A and B held the intervention with breast cancer patients 1–5. SCN C and D held the intervention with one simulated breast cancer patient, who acted out four different patient roles, resulting in breast cancer patients 6–9 (Table 3 and Figure 3). The simulated breast cancer patient was a volunteer actor who works regularly with the School of Medicine at the University of St Andrews to facilitate practical and communication training with medical students. They assumed the persona of several FCR case studies, developed by GMH based on previous real clinical cases. Nine participants were chosen as this study sought sufficient complexity in the transcripts of intervention discussions to ensure all aspects of the FOI measure could be tested.

**TABLE 3:** Breast cancer patients’ eligibility criteria.

Inclusion criteria	Exclusion criteria
Moderate FCR as defined by the FCR4 (Humphris et al., 2018)	Low (< 60th percentile) or high (> 90th percentile) FCR as defined by the FCR7 (Humphris et al., 2018)
Completed primary cancer treatment	Not yet completed their cancer treatment
Cancer-free	Not cancer-free
	Major psychological disorder(s)
	Male

#### Data Collection

The Mini-AFTERc intervention was delivered through one single telephone conversation between SCNs and NHS breast cancer patients. Additionally, four simulated telephone conversation between SCNs and the simulated breast cancer patient were recorded as part of SCNs training for the Mini-AFTERc pilot trial (McHale et al., 2020). All SCNs and NHS breast cancer patients were consented to participate. The conversations were audio-recorded using Tascam DR-05X, resulting in good audio quality. Audio-recordings were brought to the School of Medicine, University of St Andrews for storage and analysis. The data from all SCNs were included to ensure the level of fidelity was not due to a specific therapist technique used by one SCN.

#### Thematic Framework Analysis

**FIGURE 4:**
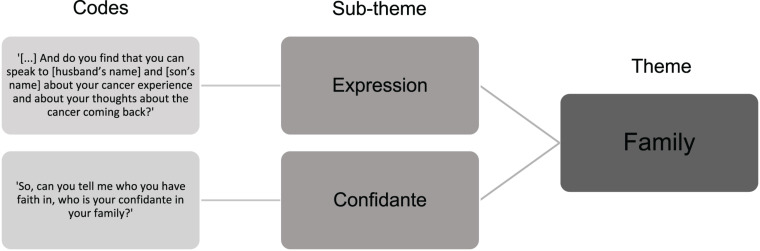
Example of codes sorted into the relevant sub-theme and theme of the Mini-AFTERc intervention.

Given that clearly defined themes already exist in the Mini-AFTERc intervention (Figure 1), a deductive, analyst-driven approach was used. This approach allows existing theoretical concepts to be brought in that provide a basis for “seeing” the data (Braun and Clarke, 2006, 2012). As the intervention’s components were discussed explicitly and implicitly, the data was approached semantically and latently (Braun and Clarke, 2006, 2012). All nine audio-recorded intervention discussions were transcribed verbatim (total duration = 254.04 min). The author reviewed the transcripts against the audio-recordings several times to ensure accuracy and correct potential mistakes (Terry et al., 2017). To test the FOI measure, the transcripts were analyzed according to the thematic analysis principles outlined by Braun and Clarke (2006). This process involved familiarization with the data, such as transcribing the audio-recordings, reading and re-reading through the transcripts and taking initial notes. Data coding was completed by highlighting particular sentences or phrases in different colors to represent different codes. Lastly, the different codes were sorted into the relevant, clearly defined sub-themes and themes of the Mini-AFTERc intervention (Figure 4).

#### Ethical Considerations

Ethical approval for this study was granted for the Mini-AFTERc pilot trial by the NHS Research Ethics Committee (18/SS/0135) and the University of St Andrews Teaching and Research Ethics Committee (MD14241). All nurse and patient participants provided written consent to participate. Ethical issues were considered by removing all identifiable information from the transcripts, including patients’ names, family’s or friends’ names, and names of locations, places, or organizations.

### Study Objective 3: Testing the Consistency of the Mini-AFTERc FOI Measure Using Interrater Reliability

NGB listened to the audio-recordings available and read verbatim transcripts; two researchers, who are considered experts of the Mini-AFTERc intervention (originator and feasibility investigator: GMH and CTM, respectively) read verbatim transcripts to rate the SCNs’ adherence to the intervention manual independently of each other.

Interrater reliability analysis was performed by NGB on nine transcripts using intraclass correlation on IBM SPSS Version 24 on macOS Mojave Version 10.14.1. The intraclass correlation coefficient (ICC) estimates and their 95% confident intervals (CI) were calculated based on mean-rating (*k* = 3; k is the number of raters), consistency, and a two-way mixed-effects model, for the total fidelity score of each of the nine transcripts. Interpretation was as follows: < 0.50, poor; 0.50–0.75, moderate; 0.75–0.90 good; > 0.90, excellent (Koo and Li, 2016; Perinetti, 2018).

## Results

### Study Objective 1: Development of the Mini-AFTERc FOI Measure, Including the Design of a Scoring System and Rating Criteria

See Supplementary File 2 for the full FOI measure. A description of the development process can be found in “Materials and Methods” section of this paper.

### Study Objective 2: Content Validating the Mini-AFTERc FOI Measure Using Thematic Framework Analysis

The nine analyzed conversations lasted between 12:42 and 45:20 min (*M* = 28:18 min, *SD* = 9.00). The average total fidelity score across all evaluated intervention discussions was 27 (range: 19–34), reflecting moderate adherence to the intervention manual. The qualitative findings indicated that the Mini-AFTERc FOI measure has content validity as it was able to measure all five components of the Mini-AFTERc intervention: Introduction, assessment, main topic(s), conclusion, and duration. Additionally, the subject matter experts, GMH and CTM, judged the contents of the FOI measure to be relevant and representative to those of the Mini-AFTERc intervention. The qualitative findings including examples of, and explanations for, FOI ratings are presented in the following sections.

#### Introduction (Setting the Scene)

The total fidelity score ranged between 3 and 7 among SCNs out of a possible 8. SCN_D_ held two interventions and was rated above average, as they exhibited personal attributes such as being warm, friendly, and supportive (Table 4). In contrast, SCN_C_ held two interventions but did not receive a score above average, mostly because they did not address subcomponents b and d in both interventions held, which led to a rating of 0 for both. Additionally, SCN_C_ received a rating of 1 for subcomponent c, as it was unclear what questions they were referring to, and what the intervention will be about (Table 4).

**TABLE 4:** Fidelity of implementation ratings for the “introduction (setting the scene)”.

Subcomponents	Supporting quotes by SCNs	FOI rating	SCN (Patient)
a. Introduction of the SCN to the patient	‘Hello [patient name], it’s [SCN’s name], [SCN’s name] one of the breast care nurses at the [place].’	2	C (7)
	‘Hello. It’s [SCN name].’	1	A (3)
b. The SCN thanks the patient for partaking in this discussion	‘Can I first of all thank you very much for agreeing to take part?’	2	A (2)
c. The SCN tells or reminds the patient of the reason for having this discussion and what it will be about	‘You filled in a questionnaire about breast cancer coming back and you rated your concerns a little bit higher. That’s why we are having a chat about your concerns and hopefully we’ll be able to help you answer some of the questions and kind of alleviate some of the concerns you have.’	2	B (4)
	‘And um, you had received the, the, the letter about the study they’re doing and um, [patient’s name] had went through some paper work and some, some questions and felt this would be a good study for you?’	1	C (7)
d. The SCN gives the patient a vague indication of what they can hope to get out of this discussion	‘So hopefully you know after today’s conversation, hopefully we’ll be able to help you, you know, feel as so you can cope with these things a wee bit, so hopefully you’ll find some benefit from it.’	2	D (8)
	Not addressed	0	B (5)

#### Assessment of Which Topics Require Detailed Discussion

The SCNs’ total fidelity score ranged between 4 and 8 out of a possible 8. As SCN_A_ had technical difficulties with the tape recording, they received an average total fidelity score of 4. All SCNs exhibited personal attributes and used therapist techniques (e.g., reflection; Table 5) essential for therapeutic alliance. For example, SCN_C_ used therapist techniques, such as exploration and facilitating the expression of affect, which reflects their high score (Table 5, a). In contrast, SCN_A_ did not attend to the patient’s experience, which reflects their moderate score (Table 5, c).

**TABLE 5:** Fidelity of implementation ratings for the “assessment”.

Subcomponents	Supporting quotes by SCNs and patients	FOI rating	SCN (Patient)
a. Family	N: ‘And do you find that you can speak to [husband’s name] and [son’s name] about your cancer experience and about your thoughts about the cancer coming back?’	2	D (8)
	N: ‘And did you feel that you were able to talk to [friend’s name] about this? Was she, was she quite supportive or did you feel you could not ask her things cause you were being protective about her?’	2	C (6)
b. Thoughts and Feelings	N: ‘How about your thoughts and feelings? Do you worry about having any aches or pains? How do you manage it? […] When you have sort of an ache or a pain, which is a bit different, how do you feel? How do you cope with that? How do you react?’	2	B (4)
c. Expectation	N: ‘So, do you think because of these fears, are you examining yourself more?’	2	C (7)
	N: ‘And hopefully when you have your mammograms and come back to the clinic, you will get that reassurance that everything’s ok. When you come to the clinic for a mammogram, how does that make you feel?’	2	B (5)
	N: ‘Do you?’ P: ‘Yes, I do. I have more time to think about it now as I live on my own, I suppose, but [friend’s name] seems very good. I don’t say much to my family at all. They have a hard time accepting everything. They are a bit worried that the cancer comes back, and I don’t say too much to them. My sister is the only one I can [confide in]. My friends are great, but we don’t actually talk about it. It’s just [friend’s name] that talked about it, because unfortunately [friend’s name] […] is going through exactly the same thing. […] She is doing the radiotherapy. But hers didn’t come back. It was the first time she had [cancer].’ N: ‘Oh, dear. Just tell me how do you feel when the annual review comes up? When your check-up comes around?’	1	A (1)
d. Return of cancer	N: ‘Um, I’m just wondering about you know, if you’ve, how are things getting, do you feel things are getting back to normal, have you gone back to work at all? […] Have you done anything you know nice like book yourself a wee holiday or anything?	2	D (9)

#### Topic of Which Specific Attention Is Required

Each of the four components that form the main part of the intervention were present in the transcripts. The findings are presented in the following subsections.

##### Family

Four interventions held by two SCNs covered this component as the main topic of the intervention. The total fidelity score ranged between 5 and 11 out of a possible 12. SCNs used therapist techniques such as exploration and reflection, and had personal attributes, such as being warm, friendly and interested, which reflect their high scores (Table 6). For example, SCN_D_ reflected back the patient’s words to explore whether the patient felt the need to be protective of their family members, whilst being open and interested (Table 6, e).

**TABLE 6:** Fidelity of implementation ratings for the main topic “family”.

Subcomponents	Supporting quotes by SCNs and patients	FOI rating	SCN (Patient)
a. Information on family	N: ‘Can you explain to us who your confidante is, in whom you’re going to when you’re worried about things?’ P: ‘Any family member.’ N: ‘Family? What family members do you have?’ P: ‘Mother in law, sisters, sisters in law, friends as well.’ N: ‘And your daughter lives with you?’	2	A (2)
b. Previous experience of cancer	N: ‘Just to go back to something you said at the beginning. You said that it was quite difficult because everybody that you knew had breast cancer.’ P: ‘Right.’ N: ‘So quite a negative experience […] I think it’s actually difficult to see that when you’re surrounded by so many people that have had the disease come back.’	2	A (3)
c. Expression	N: ‘Oki doke. And do you find that you can speak to [husband’s name] and [son’s name] about your cancer experience and about your thoughts about the cancer coming back?’	2	D (8)
d. Family supportive or antagonistic	N: ‘I’m sure when you worry about things you probably feel that you have to protect her [daughter] a little bit. Have you got somebody that you feel you don’t have to protect? A sister or your mum?’ P: ‘Yeah’ N: ‘Can you be quite open with them?’ P: ‘Yes.’ N: ‘So, it is a safe place to talk to them. And they are quite supportive?’	2	A (2)
e. Protective	N: ‘Aha, ok, ok. Um, just going back a wee bit, I appreciate what you were saying about your husband [husband’s name] and that’s wonderful to hear how supportive he’s been um, sometimes and I don’t know whether this is how you feel but sometimes patients feel as though they got to put on a good face, and they’ve got to be positive as do their loved ones and you sometimes feel that you don’t want to be a burden to people? Do you feel like that at all?’	2	D (8)
f. Confidante	N: ‘So, can you tell me who you have faith in, who is your confidante in your family?’ P: ‘My husband.’ N: ‘Your husband! Do you feel that you have to protect him, or can you really go and tell him how you’re really feeling?’	2	A (3)

##### Thoughts and Feelings

Four interventions held by three SCNs covered this component. The total fidelity score ranged between 5 and 8 out of a possible 8. All SCNs had personal attributes, such as being open and flexible, and used therapist techniques such as exploration, attending to the patient’s experience and accurate interpretation during their conversation with the patient, which reflect their high scores (Table 7).

**TABLE 7:** Fidelity of Implementation ratings for the main topic “thoughts and feelings”.

Subcomponents	Supporting quotes by SCNs and patients	FOI rating	SCN (Patient)
a. Vigilance level	N: ‘Are you paying more attention to symptoms or sensations in your body?’	2	C (7)
b. Consequences	N: ‘So, when you, you’re saying that when you get shoulder pain, sounds like you automatically get into thinking oh my goodness is it the cancer coming back.’ P: ‘Yes.’ N: ‘And is the shoulder pain there all the time?’ P: ‘No, no. Over the last couple of weeks, it’s maybe happened maybe five times or something but just kind of, uh, it just kind of I mean it’s possibly nothing to do with it at all. I suppose it just being my left, the left side of me uh, it, in kind of my more rational moments I think it’s nothing to do with anything, and then when it’s there I’m thinking oh my god what’s this?’	2	C (6)
c. Anxiety	N: ‘M hm, and before all this were you the kind of person that worried about things or is this…?’	2	C (7)
d. Coping	N: ‘[…] When you have aches and pains, do you phone the GP, or do you put it into a different perspective?’	2	B (5)
	N: ‘M hm, and what do you do when you have these feelings? And these aches and pains? Do you do anything? Do you take any painkillers or try any exercises or?’	2	D (8)

##### Expectation

Four interventions held by three SCNs covered this component. The total fidelity score ranged between 4 and 8 out of a possible 12. SCN_A_ showed warmth and attended to the patient’s experience, which reflects their high score (Table 8, b). Similar skills were observed for SCN_C_ who demonstrated interest, attended to the patient’s experience and used exploration as a therapist technique (Table 8, c). In contrast, although SCN_A_ accurately interpreted why the patient is checking themselves in the shower, they may have used exploration to further investigate whether there are any specific triggers to checking, which reflects their moderate score (Table 8, f).

**TABLE 8:** Fidelity of implementation ratings for the main topic “expectation”.

Subcomponents	Supporting quotes by SCNs and patients	FOI rating	SCN (Patient)
a. Annual check-up	N: ‘And have you had any further appointments recently? How do you feel before any appointments or any scans?’	2	C (6)
	N: ‘And how are you feeling about the thought of not having your, for not having any hospital appointments for a wee while, how does that make you feel?’	2	D (9)
b. Anxiety over annual check-up	N: ‘So how do you think you’re going to feel then, when your check-up comes around? […] How far away is the check, when you’re going to have it?’ P: ‘I think probably August or September.’ N: ‘So this is only July, so there’s quite a lot of worry before we get there, isn’t it?’ P: ‘Yes. […]’	2	A (2)
c. How do they check	N: ‘You’re right, you’re right. But can I ask when you’re, when you say you’re checking yourself every day in the shower, is it your breast that you’re checking, or your armpits or?’	2	C (6)
d. Checking frequency	N: ‘[…] Do you find yourself checking? Since you found your cancer came back, you’re performing self-checking? […] You do that quite often?’	2	A (1)
e. Public or private	Not addressed	N/A	N/A
f. Triggers to checking	N: ‘Every second day?’ P: ‘Yeah. I just check in the shower. It’s easier to check and this is how I found the other [lump].’ N: ‘Doing that every other day doesn’t seem unreasonable since you’re in the shower and its part of the routine.’	1	A (1)

##### Return of Cancer

One SCN discussed this component as the main topic of the intervention, with a total fidelity score of 6 out of 8. As the SCN addressed subcomponent a without attending to the patient’s experience, they received a rating of 1; although the patient directed the conversation from *Return of cancer* toward *Family*, the SCN did not attend to the patient’s experience of their mother dying from cancer and led the conversation back to the main topic *Return of cancer* (Table 9).

**TABLE 9:** Fidelity of implementation ratings for the main topic “return of cancer”.

Subcomponents	Supporting quotes by the SCN and the patient	FOI rating	SCN (Patient)
a. Patient’s opinion of recurrence likelihood	N: ‘Do you find that this stops you from planning for the future? I mean the diagnosis of cancer stops you from doing anything?’ P: ‘It’s a fear that makes you wonder if it’s going to come back. I think a lot of that has to do with my mum dying from cancer quite young, as well. My mum was 50 when she died and I’m thinking I’m 47.’ N: ‘Well, it’s not silly. Did you have the same type of cancer as your mum, or a different cancer?’ P: ‘She had ovarian cancer.’ N: ‘Ovarian, ok. But on a scale of 1 to 10 what do you measure your fear as? You know, worrying about coming back?’	1	B (4)
b. Likelihood changing	N: ‘So, as you said, you’ve done everything possible to reduce the risk […] of anything happening and you’ve taken some reassurance from that.’ P: ‘I mean it’s still. I think it’s going to come back when it’s going to come back. You do have it at the back of your mind all the time.’ N: ‘Yeah. I can understand that […]’	1	B (4)
c. Consequences of recurrence	N: ‘Have you ever thought what […] you know, what do you think would happen if it [cancer] did come back?’	2	B (4)
d. Future planning	N: ‘Do you find that this stops you from planning for the future? I mean the diagnosis of cancer stops you from doing anything?’	2	B (4)

##### Conclusion

The total fidelity score ranged between 1 and 4 among SCNs out of a possible 6. SCN_D_ received a rating of 2, as they showed interest and used exploration as a therapist technique (Table 10, c). In contrast, SCN_C_ received a rating of 1 as they could have been more open and interested in the patient getting a benefit out of the intervention discussion (Table 10, c). Compared to other parts of the intervention, no SCN addressed all three subcomponents of the Conclusion.

**TABLE 10:** Fidelity of implementation ratings for the “conclusion”.

Subcomponents	Supporting quotes by SCNs	FOI rating	SCN (Patient)
a. The SCN asks whether there is anything else the patient would like to discuss	‘Ok was there anything else that you wanted to talk about just now […]?’	2	D (8)
	Not addressed	0	B (4)
b. The SCN thanks the patient for attending the session	‘[…] but thank you for, um, agreeing to do this […] thank you [patient name], […]’	2	C (7)
	‘Thank you for taking part in that.’	1	B (5)
	Not addressed	0	D (8)
c. The SCN states that they hope the patient got some benefit out of this discussion and that it may have helped them a little	‘M hm, and do you feel there’s been anything that’s been helpful with the conversation?’	2	D (9)
	‘[…] hope you found it quite helpful.’	1	C (7)
	Not addressed	0	A (2)

##### Duration

The total fidelity score ranged between 0 and 2 out of a possible 2 among SCNs. SCN_A_ held the intervention with three patients and received different scores for the duration for each of them: 0, 1, and 2, for 12:42 min, 23:31 min, and 27:33 min, respectively. In contrast, SCN_D_ held two interventions with a simulated patient and received a score of 2 for both, a duration of 30:10 min and 31:40 min.

### Study Objective 3: Testing the Consistency of the Mini-AFTERc FOI Measure Using Interrater Reliability

A moderate to excellent degree of reliability was found among raters, average measure ICC = 0.84, 95% CI [0.51, 0.96]. The full interrater dataset and analysis can be found in Supplementary File 3.

## Discussion

This study developed and tested an FOI measure, designed specifically for the Mini-AFTERc intervention to assess and categorize adherence to the intervention manual. The findings indicate that, through the processes of development outlined, this FOI measure is a useful and practical tool to comprehensively assess the delivery of the Mini-AFTERc intervention, that it has content validity, and that it is reliable with the sample used in this study.

### Mini-AFTERc FOI Measure: Scoring System and Rating Criteria

The 3-point scoring system was found to be useful in practice, as it allowed researchers to employ their discriminative abilities (Jacoby and Matell, 1971) and reduced the possibility of introducing error. Therefore, the reduced rating scheme from nine to three categories can be considered more precise and accurate compared to complex scoring systems with various response categories. Using therapist principles that positively impact on therapeutic alliance (Ackerman and Hilsenroth, 2003) as rating criteria were instructive and facilitated the rating process, allowing a more accurate distinction to be made between high and moderate adherence. Consequently, these categorical definitions for each rating point may increase consistency among prospective raters who are unfamiliar with this FOI measure.

### Content Validating the Mini-AFTERc FOI Measure: Qualitative Findings

The qualitative findings indicated that the Mini-AFTERc FOI measure has content validity as it was able to measure all five components of the Mini-AFTERc intervention: Introduction, assessment, main topic(s), conclusion, and duration. Additionally, the subject matter experts judged the contents of the FOI measure to be relevant and representative to those of the Mini-AFTERc intervention, allowing valid results to be produced. Consequently, the FOI measure’s scores may be used to make important and relevant implications, suggestions, and interpretations, and also allow researchers to link the FOI results to the intervention’s effectiveness and thus draw accurate conclusions. As mentioned previously, existing FCR intervention studies do not consistently address the development and testing of FOI, which may have implications for how the results of these interventions are reported and interpreted (Lebel et al., 2014; Dodds et al., 2015; Dieng et al., 2016; Butow et al., 2017; van de Wal et al., 2017; Tomei et al., 2018). Although many of these studies do employ fidelity assessment tools, they are often not comprehensively described and/or their development and implementation is not addressed. Therefore, it is unclear whether many of these fidelity tools have content validity and whether their results accurately reflect treatment fidelity. This lack of clarity may result in inaccurate application of the fidelity tools, ambiguity regarding the integrity of the tools, and inaccurate conclusions about the efficacy of the intervention. This paper provides a detailed report of how FOI was conceptualized, developed and tested for the Mini-AFTERc intervention to ensure content validity, which may provide a template for future FCR intervention fidelity assessment tools. We propose that the systematic and evidence-based development of an FOI measure, as well as transparent, detailed and replicable reporting of such measures, should be the norm within FCR intervention research, as it is within many other areas of psychological intervention research. We hope that the work detailed in this paper will have a wider impact in that it will provide a rigorous methodological and analytical template for other researchers seeking to develop bespoke fidelity measures of healthcare communication interventions. Such practices would improve clarity, reduce bias and inform and facilitate improved FOI tool development. Most importantly, robust FOI development and assessment may lead to the refinement of FCR interventions and ensure their effectiveness for clinicians and patients.

### Testing the Consistency of the Mini-AFTERc FOI Measure Using Interrater Reliability

The results indicate a moderate to excellent consistency among the three raters, as per the classification from Koo and Li (2016). However, there was noticeable total fidelity score variability among raters, which may be attributable to the rating process. NGB listened to the four audio-recordings available to transcribe the data and rate the intervention discussions. In contrast, GMH and CTM rated the intervention discussions using the transcripts only. Research has reported that transcribing spoken data loses information as emotional responses or concrete events are translated into written language; this results in the loss of the fundamentals of natural speech, such as intonation and stress, which increase insights and understanding beyond the explicit content (Markle et al., 2011). As the rating process involved consideration of the principles of therapeutic alliance to differentiate between moderate and high adherence, GMH and CTM may have had less information on which to base and interpret their ratings and scorings of these principles, as opposed to NGB. This may explain some of the scoring variability.

### Strengths, Limitations, and Future Research

A strength of the present study is the unambiguity of the 3-point scoring system. This allows raters to employ their discriminative abilities, increases accuracy and limits the possibility of introducing error (Jacoby and Matell, 1971) as opposed to the 9-point scoring system designed during the initial development of this FOI tool. A further strength is the variety of audio-recordings and transcripts, such that each of the core components of the Mini-AFTERc intervention was present at least once in the intervention discussions used in this study. Consequently, this allowed the FOI assessment of all core components of the intervention. Furthermore, definitions and descriptions of the measure, its scoring system, and rating criteria make the application of the FOI measure more accessible. Finally, the moderate to excellent degree of interrater reliability among the three researchers involved in this study will allow the use of the FOI measure in research or clinical applications (Koo and Li, 2016).

A potential limitation to the application of the interrater reliability finding is that the developer of the FOI measure (NGB) was involved in rating the transcripts and provided instructions and explanations face-to-face to the other two raters. Therefore, future research should establish whether similar levels of interrater reliability can be obtained with the use of written and/or video instructions, that could be distributed to researchers who are unfamiliar with the FOI measure. Furthermore, it is commonly accepted that data should be collected from a broad range of participants to obtain accurate estimates of reliability in research. However, the sample of this study was relatively small due to restricted resources. Nevertheless, this study yielded important qualitative and quantitative findings, and future research should consider replicating the current study with a larger sample of patients, SCNs, and raters.

### Implications for Research and Practice

The availability of this unambiguous, content validated, and reliable FOI measure will allow confident application by individuals trained on how to employ this FOI measure. This FOI measure will allow researchers and clinicians to recognize what aspects of the Mini-AFTERc intervention work well or require improvement, and thus what aspects to emphasize in training sessions. To reduce the risk of potential liking bias, prospective researchers assessing the intervention’s FOI should be independent and not work directly with the SCNs holding the intervention. Lastly, the approach taken to develop the FOI measure for the Mini-AFTERc intervention may be helpful for other investigators to consider for novel interventions in the FCR field.

## Conclusion

This study developed and tested a novel FOI measure that considers the flexibility and comprehensiveness of the Mini-AFTERc intervention. The 3-point scoring system allowed raters to employ their discriminative abilities, thus increasing accuracy. The qualitative findings indicated that the FOI measure was able to assess all of the Mini-AFTERc intervention’s core components, thus indicating that the FOI measure has content validity. The quantitative findings indicated that the FOI measure has a moderate to excellent degree of reliability with the sample used. Future research should determine whether similar levels of interrater reliability can be obtained by distributing written and/or video instructions to researchers who are unfamiliar with the FOI measure, using a larger sample. Researchers trained on using the Mini-AFTERc FOI measure may use it to understand the translation of evidence-based research of the Mini-AFTERc intervention into clinical practice. The procedures outlined provide an example for other interventionists concerned with implementing FCR management programmes.

## Data Availability Statement

The datasets presented in this article are not readily available because full transcripts of Mini-AFTERc intervention discussions, used to test the FOI measure, are potentially identifiable. The data used to calculate interrater reliability are available. Requests to access the datasets should be directed to CTM, ctm2@st-andrews.ac.uk.

## Ethics Statement

This study involved human participants and was reviewed and approved by the NHS South East Scotland Research Ethics Committee 02 (18/SS/0135) and the University of St Andrews Teaching and Research Ethics Committee (MD14241). The patients/participants provided their written informed consent to participate in this study.

## Author Contributions

NGB developed the FOI measure, conducted all transcription and data analysis, and drafted the manuscript. CTM and GMH collected the audio recordings. All authors contributed to the interrater reliability scoring and edited and agreed the final manuscript.

## Conflict of Interest

The authors declare that the research was conducted in the absence of any commercial or financial relationships that could be construed as a potential conflict of interest.

## Abbreviations

Mini-AFTERc: Assessment Family, Thoughts and Feelings Expectations Return of cancer: Brief intervention
CI: Confidence Interval
FCR: Fear of cancer recurrence
FOI: Fidelity of implementation
ICC: Intraclass correlation coefficient
NHS: National Health Service
SCN: Specialist cancer nurse.
