# Supplementary material for: Development and Testing of a Novel Measure to Assess Fidelity of Implementation: Example of the Mini-AFTERc Intervention
Source: Front Psychol. 2020 Nov 25;11:601813. doi: 10.3389/fpsyg.2020.601813 (PMC7723987; doi:10.3389/fpsyg.2020.601813)
Supplement: Supplementary file 1 [file Data_Sheet_1.docx]

**Additional File 1; Definitions of the aspects of therapeutic alliance**

*Personal attributes*

*Respectful*

The interventionist is courteous and polite towards the patient. They do not act flippantly and are not judgemental of what the patient is saying.

*Flexible*

The interventionist is reactive and is able to prioritise discussion topics and change the focus of the intervention discussion easily according to what the patient is saying.

*Trustworthy*

The interventionist communicates with an appropriate positiveness and certainty, and it is evident that the patient has confidence in what they are saying.

*Warm*

The interventionist exhibits a friendly, kind, and caring demeanour toward the patient. They behave compassionately and empathically towards the patient.

*Confident*

The interventionist is clear when talking to the patient about the intervention discussion topics and effectively directs the flow of the discussion. They display certainty in their knowledge and advice to the patient and are not consistently hesitant or vague.

*Interested*

The interventionist shows a genuine interest in what the patient is saying and gives their full attention to the patient.

*Honest*

The interventionist is truthful and transparent with any advice or recommendations given to the patient.

*Open*

The interventionist is open-minded and tolerant when discussing the patient’s thoughts and experiences and encourages the patient to actively contribute to the discussion.

*Friendly*

The interventionist is pleasant, kind and helpful towards the patient. They are appropriately positive and receptive to what the patient is saying.

*Alert*

The interventionist demonstrates active listening and it is evident that they are able to identify, understand and respond appropriately to what the patient is saying.

*Therapist techniques*

*Exploration*

The interventionist seeks and finds out information about the patient, especially the issues surrounding their FCR, through open questioning and encouraging the patient to elaborate.

*Reflection*

The interventionist tries to clarify and restate what the patient says, by responding to not only the content, but also the feelings and emotions they are experiencing.

*Facilitating the expression of affect*

The interventionist is empathic and demonstrates sympathy and understanding towards expression of emotion.

*Accurate interpretation*

The interventionist reflects back their understanding of what the patient has said to ensure their interpretation of the information given by the patient is accurate.

*Attending to the patient’s experience*

The interventionist gives attention to what the patient is saying.

*Supportive*

The interventionist shows understanding and gives encouragement to the patient, providing appropriate advice and assistance as needed.

*Affirming*

The interventionist explicitly shows or states their support for the patient’s worries, fears, opinions, or ideas.

*Understanding*

The interventionist is perceptive and considerate of what the patient is saying and demonstrates compassion and empathy where appropriate.
