# Supplementary material for: Development and Testing of a Novel Measure to Assess Fidelity of Implementation: Example of the Mini-AFTERc Intervention
Source: Front Psychol. 2020 Nov 25;11:601813. doi: 10.3389/fpsyg.2020.601813 (PMC7723987; doi:10.3389/fpsyg.2020.601813)
Supplement: Supplementary file 2 [file Data_Sheet_2.docx]

**Additional File 2: Full version and description of the Mini-AFTERc FOI measure**

**Mini-AFTERc Fidelity of Implementation Measure**

This measure was developed to assess and categorise the level of fidelity of implementation (FOI) of the Mini-AFTERc intervention, which has been developed to normalise breast cancer patients’ fear of cancer recurrence.

To use this measure, firstly read the transcripts fully and repeatedly to familiarise yourself with the data, make some initial notes and follow the six procedural phases illustrated by Braun and Clarke (2006). Additionally, the step-by-step guide provided in this study should be used to ensure this FOI measure is used correctly. Thereafter, please circle the level of adherence for each subcomponent on this measure, except from part 3, where the subcomponents of a topic should only be rated if it was the major focus of the intervention (maximum two topics). Next, calculate the overall score for each part/component, before summing up the overall scores for each part/component to calculate the total score, and to categorise the level of fidelity.

The total score can be categorised into high, moderate, or low adherence as follows:

- A score between 28 and 48 reflects high adherence and thus reflects high fidelity of the nurse to the manual.
- A score between 14 and 27 reflects moderate adherence and thus reflects moderate fidelity of the nurse to the manual.
- A score between 0 and 13 reflects low adherence and thus reflects low fidelity of the nurse to the manual.

To help the researcher in rating the level of adherence, essential principles positively contributing to therapeutic alliance should be considered (Ackerman & Hilsenroth, 2003; Table A1).

Table A1. *Principles positively contributing to therapeutic alliance*

| Personal attributes | Therapist techniques |
| --- | --- |
| - Respectful | - Exploration |
| - Flexible | - Reflection |
| - Trustworthy | - Facilitates the expression of affect |
| - Warm | - Accurate interpretation |
| - Confident | - Attends to the patient’s experience |
| - Interested | - Supportive |
| - Honest | - Affirming |
| - Open | - Understanding |
| - Friendly |  |
| - Alert |  |

*Note*. Adapted from “A review of therapist characteristics and techniques positively impacting the therapeutic alliance” by Ackerman, S. J. and Hilsenroth, M. J., 2003, *Clinical Psychology Review, 23*(1), 28.

- If the SCN displays personal attributes *and* uses therapist techniques during the intervention as shown in Table 1, the SCN should receive a rating indicating high adherence (2).
- If the SCN does not display personal attributes *and* uses therapist techniques during the intervention as shown in Table 1, and does not consider the intervention’s flexibility, they should receive a rating indicating moderate adherence (1); this includes adhering too strictly to the manual and not taking into account possible issues that had been shared previously in the interaction by the patient.
- A rating of 0 is attributed to a component that is not addressed.
- The duration should be rated as 2 if it was within the limits of 25-35 minutes, 1 if it was within 20-40 minutes, and 0 if it was below 20 minutes or above 40 minutes.
- No negative points should be given for flexibility; when rating the main topic of the intervention, points should be given for any subcomponents of the main topic already discussed during the *Assessment* process.

Table A2. *Mini-AFTERc Fidelity of Implementation Measure*

| Part no., Components, and Subcomponents | Delivery of the Mini-AFTERc intervention | | |
| --- | --- | --- | --- |
|  | High adherence | Moderate adherence | Low adherence |
| Part 1: Introduction |  | | |
| 1. Introduction of the nurse to the patient | 2 | 1 | 0 |
| 1. The nurse thanks the patient for partaking in this discussion | 2 | 1 | 0 |
| 1. The nurse tells or reminds the patient of the reason for having this discussion and what it will be about | 2 | 1 | 0 |
| 1. The nurse gives the patient a vague indication of what they can hope to get out of this discussion | 2 | 1 | 0 |
| Overall score for part 1: |  | | |
| Part 2: Assessment by the nurse of which topics require detailed discussion |  | | |
| 1. *Family*: e.g. previous experience of cancer through family or friends? | 2 | 1 | 0 |
| 1. *Thoughts and Feelings*: e.g. determine patient’s interpretation of symptoms | 2 | 1 | 0 |
| 1. *Expectation*: e.g. explore self-examination and checking behaviour | 2 | 1 | 0 |
| 1. *Return of cancer*: e.g. determine concerns about future and remaining well | 2 | 1 | 0 |
| Overall score for part 2: |  | | |
| Part 3: Topic of which specific attention is required***** |  | | |
| 1. Family |  | | |
| 1. Information on family: Acquire a brief outline of the family composition | 2 | 1 | 0 |
| 1. Previous experience of cancer: Explore whether the patient has previous experience of cancer through a family member or a friend, and if so, what affect it has on their own experience | 2 | 1 | 0 |
| 1. Expression: Ascertain whether the patient discusses the possibility of cancer recurrence and their concerns surrounding this, with family/friends | 2 | 1 | 0 |
| 1. Family supportive or antagonistic: Ascertain whether family relationships are supportive or antagonistic to the patient in their continuing care following primary treatment | 2 | 1 | 0 |
| 1. Protective: Ascertain whether patients feel they need to protect family members of the burden of having a relative with cancer, and the level of protective feelings | 2 | 1 | 0 |
| 1. Confidante: Identify whether the patient has a confidante that they can share their condition and recovery with | 2 | 1 | 0 |
| Overall score for part 3.1: |  |  |  |
| 1. Thoughts and feelings |  |  |  |
| 1. Vigilance level: Patients with FCR are hyper-vigilant to new bodily sensations and symptoms; these need to be elicited in the discussion | 2 | 1 | 0 |
| 1. Consequences: If the patient experiences symptoms that trigger recurrence fears, may assess the extent of their reaction to these experiences | 2 | 1 | 0 |
| 1. Anxiety: Assess whether the patient is generally relatively anxious | 2 | 1 | 0 |
| 1. Coping: Investigate the patient’s coping mechanisms | 2 | 1 | 0 |
| Overall score for part 3.2: |  |  |  |
| 1. Expectation |  |  |  |
| 1. Annual check-up: Establish when the patient’s annual review is and how they feel about it | 2 | 1 | 0 |
| 1. Anxiety over annual check-up: If the patient is anxious about their annual check-up, explore how long it is before check-up that they start to feel anxious, and explore why it is causing them anxiety | 2 | 1 | 0 |
| 1. How do they check: Ascertain how the patient is checking themselves | 2 | 1 | 0 |
| 1. Checking frequency: Determine the frequency of these checking behaviours, be it scan, self-check, or otherwise | 2 | 1 | 0 |
| 1. Public or private: Ascertain whether the patient shares the result of a self-examination with anybody, e.g. friends or family (why?) | 2 | 1 | 0 |
| 1. Triggers to checking: Establish if there are particular triggers that cause the patient to self-check or schedule a scan | 2 | 1 | 0 |
| Overall score for part 3.3: |  |  |  |
| 1. Return of cancer |  |  |  |
| 1. Patient’s opinion of recurrence likelihood: Ask the patient if they believe the cancer is likely to return, e.g. ask patient to score the likelihood of recurrence on a 0-10 scale | 2 | 1 | 0 |
| 1. Likelihood changing: Assess whether the patient’s belief that the cancer will return is constant or fluctuates | 2 | 1 | 0 |
| 1. Consequences of recurrence: Determine what the patient believes the consequences of having a recurrence would be | 2 | 1 | 0 |
| 1. Future planning: Explore whether the patient makes plans for the future | 2 | 1 | 0 |
| Overall score for part 3.4: |  |  |  |
| Part 4: Conclusion |  |  |  |
| 1. The nurse asks whether there is anything else the patient would like to discuss | 2 | 1 | 0 |
| 1. The nurse thanks the patient for attending the session | 2 | 1 | 0 |
| 1. The nurse states that they hope the patient got some benefit out of this discussion and that it may have helped them a little | 2 | 1 | 0 |
| Overall score for part 4: |  |  |  |
| Part 5: Duration |  |  |  |
| 1. The nurse adheres to the approximate duration of 30 minutes (range of 25 minutes to 35 minutes) | 2 | 1 | 0 |
| Overall score for part 5: |  |  |  |
| **Total FOI score** |  |  |  |

Note. *only rate the topic if discussed in greater detail, i.e. it was the major focus of the intervention, that is, if two out of four subcomponents were discussed, or three out of six subcomponents.
