# Supplementary material for: Development and Testing of a Novel Measure to Assess Fidelity of Implementation: Example of the Mini-AFTERc Intervention
Source: Front Psychol. 2020 Nov 25;11:601813. doi: 10.3389/fpsyg.2020.601813 (PMC7723987; doi:10.3389/fpsyg.2020.601813)
Supplement: Supplementary file 3 [file Data_Sheet_3.docx]

**Additional File 3: Interrater data and analysis**

| Transcript | Rater 1  (NGB^a^) | Rater 2  (GMH^b^) | Rater 3  (CTM^c^) |
| --- | --- | --- | --- |
| 1 | 19 | 26 | 27 |
| 2 | 31 | 24 | 29 |
| 3 | 29 | 30 | 25 |
| 4 | 24 | 18 | 21 |
| 5 | 21 | 20 | 15 |
| 6 | 27 | 36 | 24 |
| 7 | 23 | 18 | 17 |
| 8 | 34 | 36 | 34 |
| 9 | 29 | 25 | 25 |

*Note*. ^a^Developer of the FOI measure. ^b^Originator of the Mini-AFTERc intervention. ^c^Feasibility investigator.

Relationship between the total fidelity scores of raters 1 (NGB) and 2 (GMH) on the FOI measure for the nine transcripts of intervention discussions. The vertical and horizontal axis represent the scale range of the total fidelity score (0-48). Average measure ICC = .71, 95% CI [-0.29, 0.94].

Relationship between the total fidelity scores of raters 2 (GMH) and 3 (CTM) on the FOI measure for the nine transcripts of intervention discussions. The vertical and horizontal axis represent the scale range of the total fidelity score (0-48). Average measure ICC = .82, 95% CI [0.18, 0.99].

Relationship between the total fidelity scores of raters 1 (NGB) and 3 (CTM) on the FOI measure for the nine transcripts of intervention discussions. The vertical and horizontal axis represent the scale range of the total fidelity score (0-48). Average measure ICC = .82, 95% CI [0.19, 0.96].
